# A random survival forest illustrates the importance of natural enemies compared to host plant quality on leaf beetle survival rates

**DOI:** 10.1186/s12898-018-0187-7

**Published:** 2018-09-10

**Authors:** Thomas A. Verschut, Peter A. Hambäck

**Affiliations:** Department of Ecology, Environment and Plant Sciences, Stockholm University, 106 91 Stockholm, Sweden

**Keywords:** *Galerucella sagittariae*, Host selection, Leaf nutrients, Natural enemy, Oviposition, Parasitism, Predation, Random survival forest, Wetlands

## Abstract

**Background:**

Wetlands are habitats where variation in soil moisture content and associated environmental conditions can strongly affect the survival of herbivorous insects by changing host plant quality and natural enemy densities. In this study, we combined natural enemy exclusion experiments with random survival forest analyses to study the importance of local variation in host plant quality and predation by natural enemies on the egg and larval survival of the leaf beetle *Galerucella sagittariae* along a soil moisture gradient.

**Results:**

Our results showed that the exclusion of natural enemies substantially increased the survival probability of *G. sagittariae* eggs and larvae. Interestingly, the egg survival probability decreased with soil moisture content, while the larval survival probability instead increased with soil moisture content. For both the egg and larval survival, we found that host plant height, the number of eggs or larvae, and vegetation height explained more of the variation than the soil moisture gradient by itself. Moreover, host plant quality related variables, such as leaf nitrogen, carbon and phosphorus content did not influence the survival of *G. sagittariae* eggs and larvae.

**Conclusion:**

Our results suggest that the soil moisture content is not an overarching factor that determines the interplay between factors related to host plant quality and factors relating to natural enemies on the survival of *G. sagittariae* in different microhabitats. Moreover, the natural enemy exclusion experiments and the random survival forest analysis suggest that natural enemies have a stronger indirect impact on the survival of *G. sagittariae* offspring than host plant quality.

**Electronic supplementary material:**

The online version of this article (10.1186/s12898-018-0187-7) contains supplementary material, which is available to authorized users.

## Background

It has become increasingly clear that the composition of terrestrial arthropod communities results from the interplay between trophic and environmental processes [1–3]. While recent studies have highlighted how large-scale environmental gradients can affect the trophic organization of arthropod communities [4, 5], the role of local variation in regulating processes like oviposition site selection [6, 7], larval survival [8, 9] and reproductive fitness [10, 11] should not be overlooked. Despite the numerous studies that have investigated the effects of environmental heterogeneity on species distribution patterns [12–14], the relative importance of small-scale environmental heterogeneity and variation in trophic interactions remains unclear, in particular because few have studies addressed them simultaneously [15–17].

Wetlands and marshy riversides are examples of habitats where local variation in water and nutrient availability results in small-scale differences in the quality of host plants for herbivorous insects [18, 19]. A beneficial effect of this small-scale variation is that wetlands generally have a high plant productivity and provide profitable oviposition sites for many herbivorous insects [20, 21]. On the other hand, wetlands may also render quite unstable growth conditions depending on annual variation in precipitation [18, 22]. While arthropod community composition has been studied in various types of wetland habitats [23, 24], we still lack insight on how community composition is determined by the combined effects of local environmental variation and predation by natural enemies. We may expect considerable variation in the strength of predation from natural enemies depending on the ability of different species to survive in drier or wetter microhabitats. This is illustrated by previous studies, which suggest that wetter microhabitats are beneficial for offspring of several herbivorous insects by reducing desiccation risk [25, 26], while at the same time increasing predation risk as these habitats often attract large numbers of natural enemies [23, 24].

**Fig. 1:**
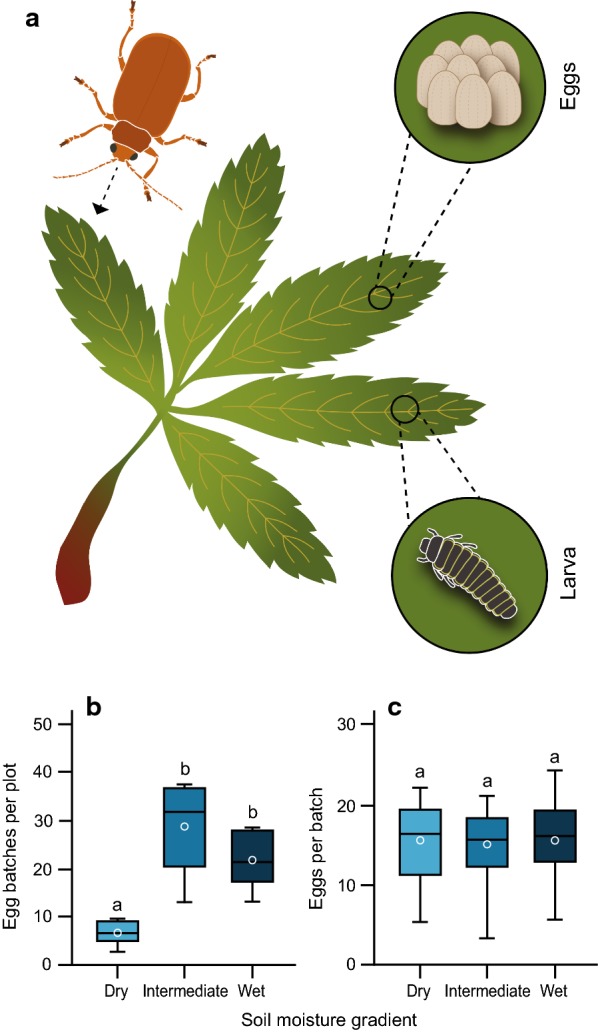
Overview of the study organisms *Galerucella sagittariae* and its host plant *Potentilla palustris.* **a** The egg batches develop for approximately 2 weeks on the lower side of the leaf, after which the larvae feed on the leaves until they pupate on the plant 3 weeks later. **b** The average number of batches per plot (n = 5) corrected for the host plant density in the plot and **c** the average number of individual eggs per batch found per plot (n = 10) in the three areas along the soil moisture gradient. The lines within the box plots represent the median and the circle represents the mean. The letters above the error bars indicate statistical differences between the areas and were calculated using Tukey-HSD post hoc analysis

We studied the relative importance of local variation in host plant quality versus predation and parasitism pressure on the offspring survival of the oligophagous leaf beetle *Galerucella sagittariae* Gyllenhaal (Fig. 1a). We had previously noticed that only a fraction of the eggs laid by this beetle survive until pupation. While we can expect that differences in host plant suitability [27], and predation pressure may both be important factors that limit offspring survival [28, 29], no previous studies have compared the effects of host plant quality and predation pressure on the survival of *G. sagittariae* offspring simultaneously. However, other studies have suggested that offspring mortality differs markedly between developmental stages and that especially early instar larvae are susceptible to predation [30]. Therefore, we quantified the variation in offspring survival along a soil moisture gradient, by following the survival of individual eggs and larvae on *Potentilla palustris* (L.) Scop, one of the main host plants of *G. sagittariae,* in a wetland dominated by this plant.

To understand the role of natural enemies, we compared natural survival rates in control plots with survival rates in natural enemy exclusion plots, in which plants were engulfed by a fine mesh to prevent predation. To identify potentially important egg and larval predators and parasitoids, we complemented our experiments by collecting potential natural enemies. We analysed the survival as time-to-event survival data using a random survival forest analysis [31, 32]. This type of analysis has gained some popularity among ecologists for their accuracy in analysing complex non-linear patterns [33, 34], and serves as a useful tool to examine an extensive collection of variables that fail to meet parametric model requirements. We hypothesized that interactions between host plant quality and natural enemy pressure varies along environmental gradients and affect the survival of eggs and larvae differently [35, 36]. This would mean that soil moisture content acts as an overarching factor on all the variables included in our model. Alternatively, it is possible that either the variables related to leaf nutrients are ranked higher, when survival is mainly affected by host plant quality, or that the vegetation structure and prey availability is ranked higher when the environmental variation mainly affected the interference of natural enemies. Ultimately, our analysis will identify which developmental stages are most sensitive to mortality, and which of the factors explain most variation in survival rates along the soil moisture gradient.

## Methods

### Study species and study location

The leaf beetle *Galerucella sagittariae* Gyllenhaal (Coleoptera: Chrysomelidae) is commonly found on various Rosaceae, Primulaceae and Polygonaceae species along lake shores, marshy riversides and wetlands in northern Europe [37]. *Galerucella sagittariae* is a univoltine species that overwinters as adults in the soil litter layer and emerges at the end of May when the host plant growing season starts. The females lay egg batches on the lower side of the leaves or on the stem until the end of June, and the eggs hatch approximately 2 weeks later, after which the larvae feed in groups on the leaves until they pupate on the plant about 3 weeks later. The eggs and larvae have been found to be predated by coccinelid beetles [28, 29], but the early instar larvae are mainly attacked by the specialist larval parasitoid *Asecodes lucens* Nees (Hymenoptera: Eulophidae) [27].

Our experiments were conducted in a marshy wetland, characterized by a strong soil moisture gradient ranging from dry soil along an elevated forest edge to water drenched soil further inwards into the wetland (Mörtsjön, Sweden—59°38′45.6″N, 18°10′03.3″E), where *G. sagittariae* exclusively uses *Potentilla palustris* (L.) Scop. (Rosaceae) as its host [27]. We identified a dry, intermediate and wet area, each approximately 30 × 30 m^2^, in a relatively straight line from the forest edge inwards into the marshy wetland where the soil moisture increased markedly. The ‘dry’ area was located next to the forest edge where the soil showed no signs of surface water. Instead, the soil was largely covered by the moss *Calliergonella cuspidata* Loeske (Amblystegiaceae), interspersed with *P. palustris* and two alternative host plants, *Lysimachia thyrsiflora* (L.) and *L. vulgaris* (L.) (Primulaceae), on which we found no signs *G. sagittariae* eggs or larvae. Approximately 100 m from the dry area we identified the ‘intermediate’ area where the soil was moist, but where the surface water only reached above the soil during long periods of precipitation. Besides *P. palustris,* the vegetation in the intermediate area also included patches of *Iris pseudacorus* (L.) (Iridaceae) and *Phragmites australis* (Cav.) Trin. ex Steudel (Poaceae). Finally, at approximately 100 m from the intermediate area we identified the ‘wet’ area in which the soil was drenched with water throughout study period. This area was dominated by *P. palustris* interspersed with a few other wetland plant species like *Carex stricta* Lam., *C. acutiformis* Ehrh. (Cyperaceae) and *Persicaria amphibia* (L.) Gray (Polygonaceae).

### Study design

At the beginning of the egg-laying season, we established fifteen 1 × 1 m^2^ plots at random locations in each of the three areas along the soil moisture gradient for a total of 45 plots. Within five haphazardly selected plots per area, we counted the total number of egg batches to estimate the abundance of egg batches at the beginning of the season along the gradient. Subsequently, we overlaid each plot with a 1 × 1 m^2^ grid, consisting of 10 × 10 cm^2^ squares, to estimate host plant density as the number of squares containing *P. palustris*. To estimate the role of neighbouring plants on offspring survival, we measured the height of the vegetation surrounding *P. palustris* host plants within each plot.

Once the vegetation was mapped, we randomly designated ten plots per area as control plots and a natural enemy exclusion treatment to the five remaining plots per area. The control plots were used to measure the survival of *G. sagittariae* offspring under natural conditions, whereas the natural enemy exclusion plots excluded any natural enemies of *G. sagittariae* offspring by enclosing plant shoots in meshed sleeves. By comparing the survival between open and enclosed plant shoots, we could separate the role of environmental and plant-related factors from the role of natural enemies on offspring survival. As *G. sagittariae* is a univoltine species, the different developmental stages do not occur at the same time. Consequently, we made egg and larval survival measurements in separate periods and treated them as separate experiments throughout the paper.

### Measuring egg survival

To estimate egg survival in the control plots, we haphazardly selected ten freshly laid egg batches per plot, counted the number of eggs per batch, and measured the height at which the batch was located on the plant. We marked each batch with small coloured plastic bands at the leaf base, and the plants with coloured sticks, to ensure that we could relocate individual egg batches. The survival of individual eggs within the batches were first counted after 6 days, followed by intervals of 4 days until all eggs had hatched or were predated. Within the natural enemy exclusion plots, we haphazardly selected four individual plants on which we located an egg batch and counted the number of eggs within those batches as previously explained. Subsequently, we enclosed the shoots with the egg batch in a 30 × 60 cm sleeve (width × length − 50 µm mesh) to exclude any natural enemies. We closed the top and bottom of the sleeves with a fine wire and attached bamboo sticks to keep the sleeves in upright position and to prevent suppression of plant growth. Once we observed that most eggs had hatched in the control plots, we opened the sleeves and counted the number of larvae as a measure of egg survival. We only counted the number of surviving eggs at the end of developmental period to prevent any possible dislocation of the eggs or destruction of the plant material while reopening and closing the meshed sleeves.

### Measuring larval survival

At the onset of larval hatching, we counted the total number of larvae present on ten individual plants, measured the height of the plant, and counted the remaining larvae every fourth day until pupation in the control plots. We only counted the larvae in the natural enemy exclusion plots at the end of the experiment to avoid disturbing the larvae when removing the mesh. The larvae were usually only found on one or two shoots, and this unit was also used in the natural enemy exclusion treatment when enclosing shoots in 30 × 60 cm sleeves (width × length − 50 µm mesh) as described for the egg survival. While *Galerucella* larvae can move to fresher leaves on the same host plant after hatching, the sprawling structured stems and pinnate leaves of *P. palustris* normally provide enough resources for the larvae to consume until pupation, and restricts larval movement to a single stem of the host plant. Our observations in the field suggest that larvae stay on the host plant on which they hatched rather than move from one host plant to another. This suggests that the number of larvae present on an individual host plant represents a relatively accurate estimate of the larval survival, even though we do not account for the unlikely event that they moved to another host plant. Because several egg batches, originally used to measure egg survival, were entirely consumed we sometimes had to select a new plant containing larvae to measure larval survival. These new plants were marked and treated following the same methods as for the original plants. Once all the larvae had pupated, we assessed whether the pupae were healthy or parasitized and used the final number of healthy pupae as a measure of larval survival.

### Natural enemy survey

During late June, we sampled the arthropod community with sticky traps, pit-falls and sweep nets to survey potential natural enemies of *G. sagittariae.* Each sampling method was repeated twice and was restricted to the vicinity of the natural enemy exclusion plots to prevent intervention with the control treatments. As sticky traps, we placed three double sided blue sheets (14 × 11 cm) covered in a strong odourless glue at an approximate height of the vegetation (20–50 cm) for 24 h next to the natural enemy exclusion plots. To complement the sticky traps, we sampled natural enemies by sweep netting through the vegetation. We standardized the sweep netting by making forty sweeping movements in the vicinity of a natural enemy exclusion plot. Finally, we placed three pit falls (⌀ 6.5 cm) filled with 70% ethanol for 24 h within the natural enemy exclusion plots. From all collected arthropods, we sorted the potential natural enemies and identified them to species level or closest taxa. Because neither of these methods captured any *A. lucens* individuals, the specialized larval parasitoid of *G. sagittariae,* we counted the number of healthy and parasitized larvae in five control plots per area at the end of the larval survival experiments. The parasitized larvae are easily separated from the healthy as they turn into hardened black mummies.

### Host plant quality

To measure variation in host plant quality, we measured the leaf moisture content (% of leaf moisture = 100 × [(Weight_*fresh *_*− *Weight_*dry*_)/Weight_*fresh*_]) and the concentrations of leaf nitrogen, carbon and phosphorus of plants along the soil moisture gradient. Shortly after sampling, we measured the fresh weight of the leaflets and then dried them at 60 °C in a drying oven for 72 h before measuring the dry weight. For the nutrient concentrations, we randomly collected three leaflets per plot and dried them at 60 °C in a drying oven for 72 h after which we ground them into a fine powder for the analysis. For the nitrogen and carbon concentration analysis, we combusted approximately 2 mg of the dried powder at 950 °C in an oxygen-rich environment to allow for organic elemental analysis (FLASH 2000 Element analyser, Thermo Scientific). The combustion products were separated in a gas chromatographic column, detected and processed by a Thermal Conductivity Detector (Eager Xperience 300, Thermo Scientific). For the analysis of the phosphorus concentration, approximately 3 mg of dried leaf powder was ignited at 500 °C in presence of MgSO_4_ and KNO_3_. The combustion products were then dissolved in an acid persulfate solution (K_2_S_2_O_8_), and the resulting phosphates were measured by segmented flow analysis (Flow Solution IV, Alpkem O.I. Analytical).

### Statistical analysis

We first corrected the number of egg batches laid per plot with the host plant density within that plot and compared the distribution of egg batches along the soil moisture gradients. Next, we compared the egg and larval survival on individual plants in control plots with that in the natural enemy exclusion plots. For both tests, we used generalized linear models (GLM) that included a random factor for the plots and a Gaussian and binomial error distributions respectively. We used the lme4 package [38] for fitting the mixed effect models and the car package [39] for likelihood ratio tests. We extended our data analysis, for the measurements made in the control plots, by performing survival analyses in which each egg or larva is included as individual right censored, time-to-event, observations. Because most survival analysis regressions require parametric data that is proportional to the risk probability [40], we first inspected the data distribution and parametric model requirements of all explanatory variables using GLMs and Tukey HSD post hoc tests using the multcomp package [41]. We designate (1) host plant height, (2) surrounding vegetation height, (3) host plant density and (4) the number of individual eggs or larvae as variables related to predator efficiency, and (5) leaf moisture content, (6) nitrogen concentration, (7) carbon concentration and, (8) phosphorus concentration as variables related to host plant quality. The analyses of individual response variables indicated that most factors did not meet the general parametric model requirements (Analysis of all variables can be found in Additional file 1: Table S1; Figure S1).

Due to this issue, we opted against using traditional survival analysis and instead analysed our data with a random survival forest (RSF) analysis using the randomForestSRC package [42] and the ggRandomForest package [43] for additional visualization of the data. RSF analysis is an extension of the random forest machine learning method of Breiman [44] to analyse time-to-event observations. This analysis offers an efficient alternative to traditional survival analysis as it does not require restrictive parametric or proportional survival assumptions [31, 32]. Based on our survival observations, we assigned right-censored survival settings to each individual egg or larvae, comprising of either a value of ‘one’ for a predation event or ‘zero’ for survival followed by the day at which the event occurred [40]. We included the untransformed data of the eight variables previously mentioned and the area along the soil moisture gradient as predictor variables in our RSF analysis (Additional file 1: Table S1; Figure S1). The RSF analysis is not a parsimonious method and instead draws a bootstrap sample out of all data and variables included in the analysis to construct survival predictions. We grew 1000 individual binary decision trees on the separate data sets for the egg and larval survival through recursive partitioning using a log-rank random splitting rule to select the optimal candidate variables [31, 32]. The analysis randomly selects ten split point values, rather than optimizing over all possible values, to split all data included at the root node into daughter nodes until a minimum of three individual observations is reached in the terminal nodes [42]. Subsequently, Kaplan–Meier survival estimators are constructed within each terminal node for all event times (i.e. time of death).

The following step of the analysis averages over the trees and builds the random forest. Approximately 36.8% of the observations are reserved for an out-of-bag (OOB) sample and used as a predictive error estimate [33, 45]. Subsequently, we used variable importance (VIMP) and minimal depth measures, calculated over the entire random forest, to identify the importance of the different variables in the survival probability of the eggs and larvae. VIMP measures are calculated by comparing the OOB data with the previously constructed survival tree. Consequently, VIMP measures the change in prediction error when the variable of interest would not be available when growing a new forest [31], while the minimal depth indicates the most important variables by determining how close to the terminal node the variables usually split the data [46]. Minimal depth offers a simple threshold rule for the importance of variables in the RSF analysis by comparing the minimal depth values of each variable with the average of the minimal depth distribution [43]. Although RSF analysis are relatively insensitive to correlation between variables [47], we used minimal depth variable interaction plots to indicate potential interactive effects with the area variable, which was our main variable of interest in terms of survival. Subsequently, we used partial dependence plots, which averages the effects of each variable over the other variables included in the forest, to visualize the risk-adjusted relation between the variables and survival probability.

To determine differences in the presence of natural enemies in the three areas, we compared the abundance per sampled species with the position along the soil moisture gradient as an explanatory variable using the adonis function of the vegan package [48]. The adonis function is a permanova that uses permutations to partition the data matrix (i.e. abundance per predator species) between areas, and allows for an accurate analysis of non-parametric abundance data. All species of which we collected less than five individuals were removed from the analysis, as they are unlikely to be common natural enemies of *G. sagittariae* offspring. Subsequently, we performed an indicator species analysis using the indval function of the labdsv package as a post hoc test [49] and used non-metric multidimensional scaling (NMDS) to quantify and visualize the compositional similarity of the species samples in the three areas along the soil moisture gradient. All analyses were carried out in R (v.3.3.2; R Foundation for Statistical Computing, Vienna, AT).

## Results

### Egg survival

**Fig. 2:**
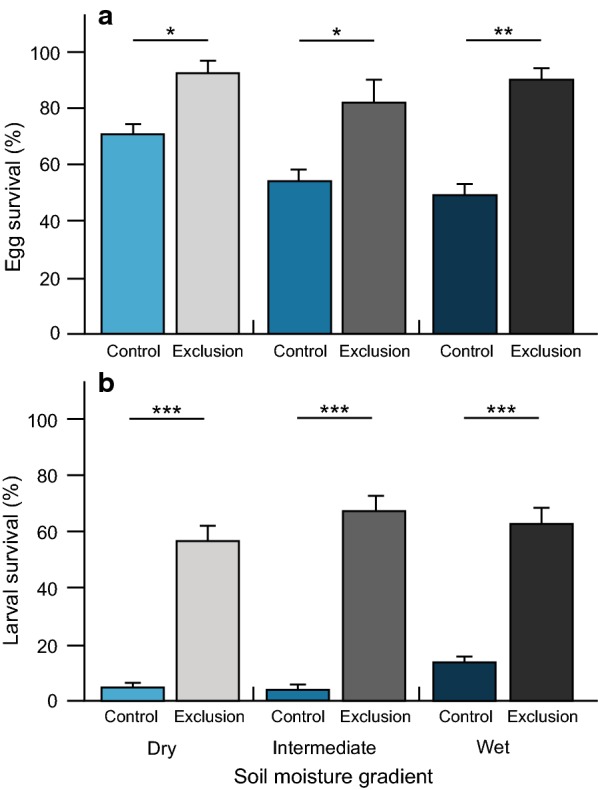
Survival of *Galerucella sagittariae* offspring in the control and natural enemy exclusion plots. The survival of individual **a** eggs and **b** larva in the control (blue) and natural enemy exclusion plots (grey) in the dry, intermediate and wet area of the soil moisture gradient. Statistical differences between the survival in the control plots and natural enemy exclusion plots were calculated through planned comparisons. *p < 0.05; **p < 0.01 and ***p < 0.001

**Fig. 3:**
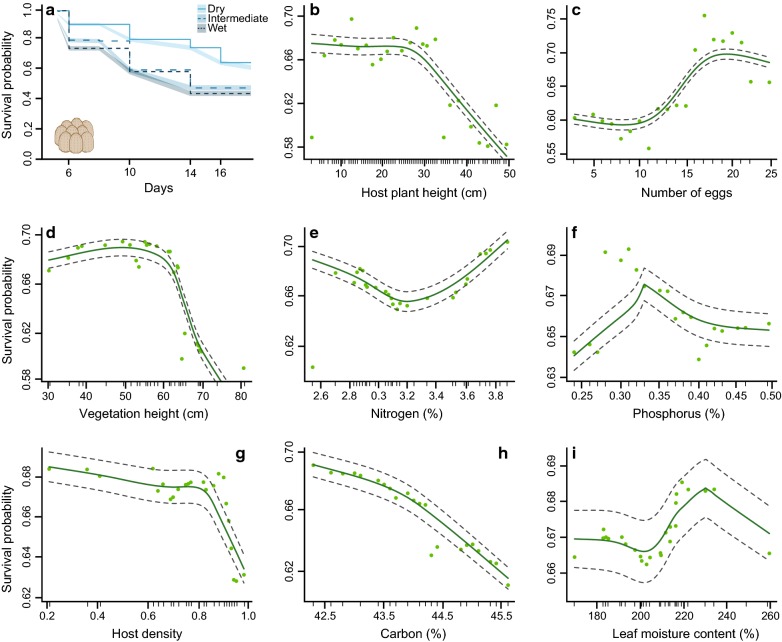
Survival probability and partial dependence plots for egg survival. **a** The survival probability of individual *Galerucella sagittariae* eggs in the dry area (solid—light blue), intermediate area (large dashed—bright blue) and wet area (small dashed—dark blue). The shaded areas give the 95% confidence intervals. All other plots give the partial dependence of all individual variables while averaging out over all variables. The dark green line corresponds to a lowess smoothed line representing the partial dependence of **b** host plant height; **c** number of eggs; **d** vegetation height; **e** nitrogen; **f** phosphorus; **g** host plant density; **h** carbon and **i** leaf moisture content on egg survival. The grey dashed lines indicate a smoothed ± two standard errors and the light green dots represent 25 of those partial values used to fit the lowess function. The small tick marks represent all values included in the random survival forest analysis

The initial number of *Galerucella sagittariae* egg batches was lower in the dry area compared to the other two areas (GLM: $\chi^{2}_{2,14}$ = 20.63, *p *< 0.001; Fig. 1b), but there was no difference in the number of eggs laid per egg batch between areas (GLM: $\chi^{2}_{2,299}$ = 3.75, *p *= 0.15; Fig. 1c; Additional file 1: Table S1). The overall egg survival was slightly lower in areas with a higher soil moisture content (GLM: $\chi^{2}_{2,356}$ = 9.11, *p *= 0.01), and was considerably higher in the natural enemy exclusion treatment compared to control plots (GLM: $\chi^{2}_{1,356}$ = 35.65, *p *< 0.001), without being affected by an interaction between area along the soil moisture gradient and treatment (GLM: $\chi^{2}_{2,356}$ = 2.01, *p *= 0.37; Fig. 2a). Additional multiple comparisons showed that the egg survival differed between the dry and wet area (Tukey HSD: *p *= 0.01), but not between the intermediate area and the two other areas (Tukey HSD: both comparisons *p *> 0.08), or between any of the areas in the natural enemy exclusion treatment (Tukey HSD: all *p *> 0.12). The random survival forest (RSF) analysis showed that the survival probability of *G. sagittariae* eggs decreased faster with time in the intermediate and wet area compared to the dry area (Fig. 3a). The comparison between the data included in the RSF analysis and the out-of-bag (OOB) data returned a predictive error estimate of 12.29% (Additional file 2: Table S2), resulting in relatively high variable importance values (VIMP), which indicate strong explanatory power of the variables included in the analysis (Table 1; Additional file 2: Table S2). Consequently, none of the variables included in the RSF analysis returned negative VIMP values (Additional file 2: Figure S2), indicating that the RSF analysis would not be improved by removing any variable (Table 1; Additional file 2: Table S3; Figure S2).

**Table 1:** Summary of the random survival forest analyses for egg and larval survival

Egg survival			Larval survival		
Variables	VIMP	Depth	Variables	VIMP	Depth
Host plant height	0.195	2.09*	Host plant height	0.022	2.29*
Eggs	0.181	2.00*	Larvae	0.016	2.12*
Vegetation height	0.131	1.86*	Vegetation height	0.016	1.76*
Area	0.109	2.16*	Area	0.015	2.11*
Nitrogen	0.087	2.25*	Host plant density	0.011	2.11*
Phosphorus	0.078	2.80	Phosphorus	0.010	2.35
Host plant density	0.079	2.62	Carbon	0.008	2.57
Carbon	0.074	2.81	Nitrogen	0.007	2.75
Leaf moisture	0.061	3.12	Leaf moisture	0.007	2.75

The variable importance is given in the VIMP column and used to rank the importance and order of the variables in this table. The minimal depth is given in the Depth column in which the * indicates minimal depth values lower than the average minimal depth over all variables of the egg survival analysis (2.41) and larval survival analysis (2.31)

Out of the top-ranking variables, we found that host plant height, the number of eggs and the surrounding vegetation height were of higher importance than the position along the soil moisture gradient (Table 1; Fig. 3 and Additional file 2: Figure S2). Moreover, the minimal depth interactivity plots indicated low interactivity between the top ranking variables and area (Additional file 2: Figure S4), suggesting that these variables did not affect the egg survival differently in the three areas. Minimal depth offers a simple threshold rule for the importance of variables by comparing them with the average of the minimal depth distribution. This comparison showed that vegetation height, number of eggs, host plant height, area along the soil moisture gradient and nitrogen were ranked as the five most important variables explaining egg survival. The overall ranking of the variables by VIMP and minimal depth was in strong agreement except for host plant height, which was ranked higher by VIMP, and surrounding vegetation height which was ranked higher by minimal depth (Table 1; Additional file 2: Figure S3). These results indicate that removing host plant height from the analysis would substantially weaken the outcome of the analysis, whereas surrounding vegetation height was better at dividing large portions of the data (i.e. splits data closer to the terminal node).

The partial dependence plots show that the egg survival probability decreased with host plant height and surrounding vegetation height (Overview analyses of all variables can be found in Additional file 1: Table S1; Figure S1), and increased with increasing egg batch size (Fig. 3). For the variables with lower importance than area, nitrogen concentration was still ranked above the average minimal depth threshold and showed a lower egg survival probability at intermediate nitrogen concentrations (Fig. 3e). Moreover, we found hump-shaped patterns for the effects of the phosphorus concentration and leaf moisture content, while increasing host densities and carbon concentrations lowered the survival probabilities (Fig. 3g, h). The minimal depth interactivity plots did not indicate interactivity between any variables ranked lower than area in the RSF analysis either (Additional file 2: Figure S4). Interestingly, the top five ranked variables did show signs of interactivity with the other variables (Additional file 2: Figure S4), but the insensitivity of RSF analyses to interactions likely minimized the effect these interactions could have on the outcome of our analysis.

### Larval survival

**Fig. 4:**
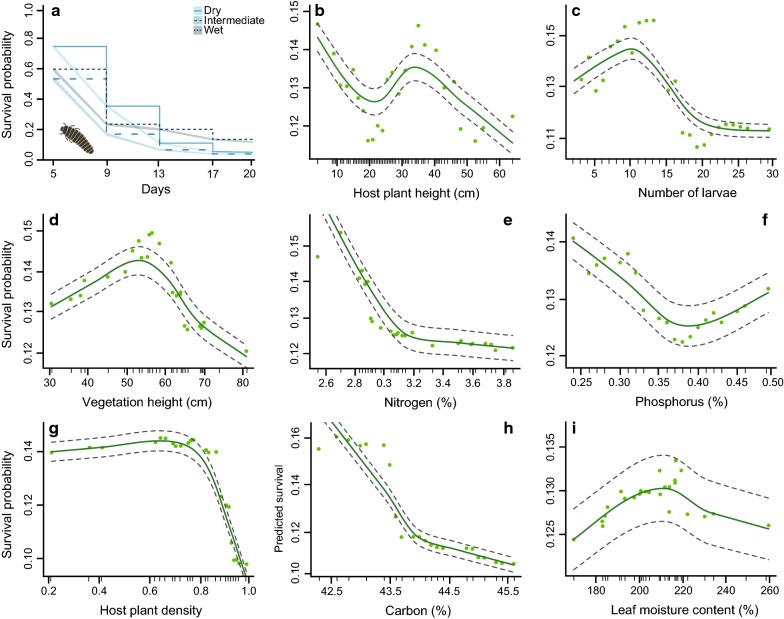
Survival probability and partial dependence plots for larval survival. **a** The survival probability of individual *Galerucella sagittariae* larvae in the dry area (solid—light blue), intermediate area (large dashed—bright blue) and wet area (small dashed—dark blue). The shaded areas give the 95% confidence intervals. All other plots give the partial dependence of all individual variables while averaging out over all variables. The variables are ordered in accordance with Fig. 3 rather than the RSF ranking (Table 1) to facilitate visual comparisons between the figures. The dark green line corresponds to a lowess smoothed line representing the partial dependence of **b** host plant height; **c** number of eggs; **d** vegetation height; **e** nitrogen; **f** phosphorus; **g** host plant density; **h** carbon and **i** leaf moisture content on larval survival. The grey dashed lines indicate a smoothed ± two standard errors and the light green dots represent to 25 of those partial values used to fit the lowess function. The small tick marks represent all values included in the random survival forest analysis

We found that larval survival was affected by the increasing soil moisture content (GLM: $\chi^{2}_{2,356}$ = 18.23, *p *< 0.001), was higher in the natural enemy exclusion treatment (GLM: $\chi^{2}_{1,356}$ = 373.74, *p *< 0.001), and was affected by an interaction between area and treatment (GLM: $\chi^{2}_{2,356}$ = 22.73, *p *< 0.001; Fig. 2b). This interaction occurred because survival increased with soil moisture content in the control plots (GLM: $\chi^{2}_{2,60}$ = 11.12, *p *= 0.003), but not in the natural enemy exclusion treatment (GLM: $\chi^{2}_{2,60}$ = 1.75, *P *= 0.42). Additional multiple comparisons showed that the larval survival in the control plots differed between the dry and wet area (Tukey HSD: *p *< 0.001), the intermediate and wet area (Tukey HSD: *p *< 0.001), but not between the dry and intermediate area (Tukey HSD: *p *= 0.81). These results were in agreement with the RSF analysis, which showed that the survival probability of the larvae decreases strongly within the first 9 days in all areas, but stabilized on a higher survival probability in the wet area compared to the two other areas (Fig. 4a). No variables included in the larval RSF analysis returned negative VIMP values (Table 1; Additional file 2: Table S2; Figure S2), but due to the low overall larval survival, the RSF analysis returned a predictive error estimate of 42.21% (Additional file 2: Table S2). This higher error estimate resulted in lower VIMP values and a lower explanatory power of the variables included in the analysis (Table 1; Additional file 2: Table S2). Consequently, while VIMP and minimal depth ranked the same five variables as the most important variables to explain larval survival, the actual ranking order of these variables was less in agreement (Table 1; Additional file 2: Figure S3). More specifically, host plant height and the number of larvae were ranked higher by VIMP, indicating that removing these variables would substantially weaken the outcome of the analysis. Furthermore, host plant density, area and surrounding vegetation height were ranked higher by minimal depth (Additional file 2: Figure S3), indicating that these variables were better at dividing large portions of the data (i.e. splits data closer to the terminal node).

We found that the host plant height, the number of larvae and the surrounding vegetation height were all of higher importance than area (Table 1; Additional file 2: Figure S2). All of these variables had low minimal depth interactivity with area (Additional file 2: Figure S5), suggesting that these variables did not affect larval survival differently along the soil moisture gradient. Interestingly, all of the variables with a higher importance than area showed a hump-shaped relationship with larval survival (Fig. 4). Similar to the RSF analysis of egg survival, the top five ranked variables did show signs of some interactivity with the other variables (Additional file 2: Figure S5). For the variables with lower importance than area, the host plant density was still ranked above the average minimal depth threshold and showed a decreasing relationship with larval survival when the density of host plants increased (Fig. 4g). For the concentrations of leaf carbon, nitrogen and phosphorus, we found negative relationships with larval survival while leaf moisture showed a hump-shaped pattern for larval survival (Fig. 4).

### Natural enemy survey

**Fig. 5:**
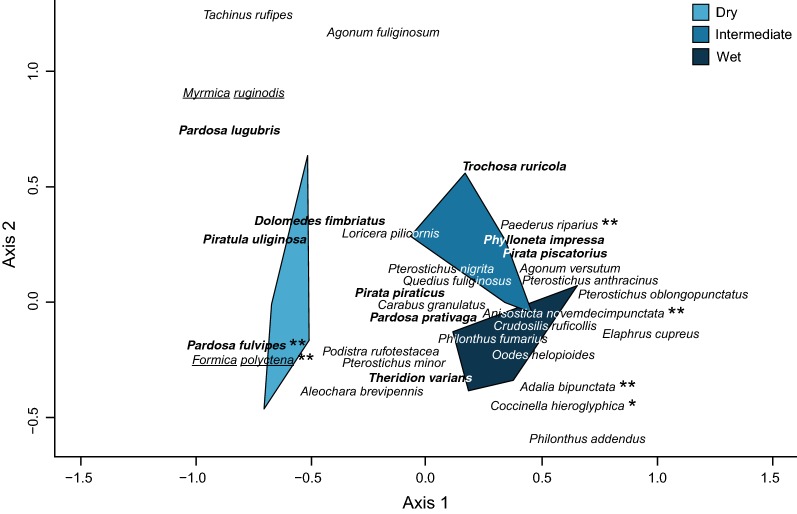
Nonmetric multidimensional scaling (NMDS) plot for the occurrence of natural enemies along the soil moisture gradient. Coleoptera are given in italic, Araneae in italic and bold, and Hymenoptera are underlined and in italic. The plot only includes those species from which we found more than five individuals, and the complete species can be found in Additional file 3: Table A3. *p < 0.05 and **p < 0.01

We identified a total of 52 potential natural enemies consisting of various Araneae, Coleoptera and Hymenoptera families (Additional file 3: Table S3). Our multivariate analysis showed that the occurrence of natural enemies differed between the three areas along the soil moisture gradient (adonis; *F*_2,12_ = 7.56, *p *= 0.001). Additionally, the indicator species analysis showed that the occurrence of *Formica polyctena* (Hymenoptera: Formicidae—*p *= 0.001) and *Pardosa fulvipes* (Araneae: Lycosidae—*p *= 0.003) were more associated with the dry area, while the occurrence of *Paederus riparius* (Coleoptera: Staphylinidae—*p *= 0.006) was more associated with the intermediate area, and the occurrence of *Anisosticta novemdecimpunctata* (Coleoptera: Coccinellidae—*p *= 0.002), *Adalia bipunctata* (Coleoptera: Coccinellidae—*p *= 0.006) and *Coccinella hieroglyphica* (Coleoptera: Coccinellidae—*p *= 0.017) with the wet area (Fig. 5). For the specialized larval parasitoid *Asecodes lucens* (Hymenoptera: Eulophidae), we found no difference in the number of parasitized larvae along the soil moisture gradient (GLM: $\chi^{2}_{2,14}$ = 1.82, *p *= 0.40; Additional file 3: Figure S6). Moreover, during the larval survival experiments we found *Acleris* sp. (Lepidoptera: Tortricidae) leaf rolling larvae on nine host plants in the dry area, 25 host plants in the intermediate area and six host plants in the wet area respectively. While these micro moth larvae might not be classified as natural enemies, their leaf rolling habits may have resulted in lower survival of *G. sagittariae* larvae on the infected host plants.

## Discussion

Our results suggest that natural enemies strongly limit the survival of *Galerucella sagittariae* offspring. While we found that egg survival decreased with increasing soil moisture content, the larval survival was low throughout the entire study area and showed a small increase with soil moisture content (Fig. 2). Furthermore, our analysis showed that the early instar larvae were by far the most sensitive developmental phase to mortality (between 1 and 9 days; Fig. 4). We hypothesized that the survival analyses would rank soil moisture content as the most important variable as it may affect both host plant quality and natural enemy density, which both are known to affect the survival of insect eggs and larvae. Interestingly, both the egg and larval survival analyses ranked host plant height, the number of eggs or larvae, and vegetation height respectively as better predictors of survival than the soil moisture gradient (Figs. 3, 4). Moreover, as our initial analyses of the response variables indicated minimal differences along the soil moisture gradient (Additional file 1: Table S1; Figure S1), our results suggest that these other variables played a more important role in determining offspring survival rates than the local variation in soil moisture content.

More specifically, our analysis showed that survival decreased non-linearly with increasing host plant- and vegetation height (Figs. 3, 4), suggesting that eggs and larvae located higher up in the vegetation are more vulnerable to be attacked. It is possible that the importance of host plant- and vegetation height on egg and larval survival is a consequence of higher densities of natural enemies in taller vegetation [50, 51], or because natural enemies find their prey easier when they are located higher up in the vegetation [52–54]. It is, for example, possible that the moist wetland soil lowered the ability of ground-dwelling natural enemies to reach prey, making canopy dwelling natural enemies pose a greater risk [55]. Moreover, predators crawling through the vegetation will likely benefit from structurally complex vegetation [50, 56], which can explain the strongly negative effect of increasing host plant density on egg and larval survival (Figs. 3, 4). We identified a diversity of canopy dwelling beetles and spiders that are likely predators in wetland communities (Additional file 3: Table S3). Among these predators, some are perhaps less likely to predate on *Galerucella* eggs and larvae. For instance, most carabid beetles have a limited capacity to climb and may be less able to predate eggs and larvae up on a plant. Spiders on the other hand are often able to climb but are less likely to predate eggs, although predation of early instar larvae may be a possibility for spiders [24]. Therefore, the most likely predators on the *Galerucella* eggs and larvae may have been the staphylinid, cantharid and coccinellid beetles, and to some extent also ants, which may all feed on prey in plant canopies and where many species are known egg and larval predators.

Among these potential predators, we found some differences in their abundance along the soil moisture gradient, which could have caused variation in offspring survival along the gradient. Our analysis indicated a more dominant occurrence of the ant *Formica polyctena* and the spider *Pardosa fulvipes* in the dry area, and a higher occurrence of the rove beetle *Paederus riparius* in the intermediate area and all coccinellid beetles in the wet area respectively. We also found a relatively general occurrence of the canopy dwelling lycosid spiders *Pirata piraticus* and *Pardosa prativaga* and staphylinid rove-beetles *Aleochara brevipennis* and *Quedius fuliginosus* (Fig. 5), but found no differences in the parasitism rate of the specialized larval parasitoid *Asecodes lucens* along the soil moisture gradient (Additional file 3: Figure S6). These results are in accordance with other studies of mortality in *Galerucella* offspring, which found high predation by ants, ground-beetles and ground-dwelling spiders in drier areas [54, 57, 58], whereas offspring in wetter areas was found to be more sensitive to predation by ladybirds [28, 29]. The high occurrence of ladybirds in the wet area could help explain the decreasing egg survival rates with increasing soil moisture content (Fig. 2), as especially *C. hieroglyphica* has been found to predate on *G. sagittariae* eggs and early instar larvae [28, 29]. Consequently, predation by coccinellid beetles may have aggravated the strong decrease in survival rates we observed for larvae in the first 9 days (Fig. 4). Furthermore, besides the rove beetles *P. riparius,* the other generally occurring rove beetle species could potentially feed on *G. sagittariae* offspring (Additional file 3: Table S5), as suggested by Wiebe and Obrycki [24].

Besides the potential effects of natural enemies, Sipura et al. [26] found a strong affinity of another *Galerucella* species to moist oviposition habitats where early instar larvae perform best. Therefore, the higher survival rates at the lower host plant heights may have also been a consequence of microhabitat quality. Firstly, desiccation is believed to be a major mortality risk for the unprotected eggs and soft bodied neonate larvae [25] and secondly, there is some evidence that larvae of other *Galerucella* species evaded the risk of being predated by developing quicker in sheltered and moist microhabitats [25, 26]. While these two microhabitat effects might have increased survival probabilities at the lower parts of the vegetation, these parts of the host plant also attracted leaf rolling *Acleris* sp. micro-moths. The moth larvae rendered the host plant useless for *G. sagittariae* larvae, and seemingly lowered the survival of *G. sagittariae* larvae in the intermediate areas. We also found some evidence that the number of conspecific eggs or larvae per plant had a strong impact on survival (Table 1). Interestingly, while eggs benefitted from larger sized egg batches (Fig. 3), larval survival was negatively affected by intraspecific competition on the same host plant (Fig. 4). While our results suggest a common fitness related consequence of lowered predation probabilities for individual eggs by increasing the egg batch sizes [59, 60], the negative effect of group size on larval survival might have two causes. Firstly, the higher number of larvae could have made it easier for natural enemies to detect them [61] and secondly, overcrowding of the leaves might have led to higher mortality rates without the interference of natural enemies [24, 62].

In our natural enemy exclusion experiments, we observed that approximately 40% of the larvae dies even in the absence of natural enemies (Fig. 2), suggesting that overcrowding, and possibly other developmental mortality causes, may significantly impact survival on host plants with high numbers of larvae. On crowded plants, desiccation sensitivity [25], and low food availability have been reported to cause high larval mortality [63], and extend their sensitivity to predation by reducing their growth rates [30]. Our results illustrate that the earliest larval instars face the highest larval mortality risks (Fig. 4a), making it possible that the lowered food availability on overcrowded plants also extended the sensitivity of *G. sagittariae* larvae to predation. Interestingly, none of the leaf nutrients or the leaf moisture content were ranked as more important than the soil moisture gradient (Table 1), suggesting that host plant quality by itself did not play a large role in survival. Especially nitrogen is assumed to be essential for developing insects, and it would be expected that higher nitrogen concentrations would be beneficial for offspring survival [64, 65]. Although nitrogen content seemed to be of some importance for egg survival, this pattern could merely have been caused by the higher nitrogen concentrations in the dry area (Additional file 1: Figure S1), where the overall egg survival probability was highest (Fig. 3). Moreover, the decreased larval survival probability with increasing nutrient concentrations could be explained by the higher nutrient concentrations in the areas where larval survival was lowest to start with.

## Conclusion

Our results suggest that host plant and surrounding vegetation heights strongly influenced offspring survival by providing beneficial microhabitats and lowering the predation rates by natural enemies. We did not find any evidence that the soil moisture gradient was an overarching factor determining the relative importance of factors relating to host plant quality versus those relating to natural enemies. Instead, we found that the wet and intermediate areas potentially offer microhabitats where desiccation risks are minimized by the higher water availability combined with the selection of the lower parts of the host plants by the egg laying female. Considering that larval stages are often the most sensitive developmental stages [30], the innate selection of microhabitats with lower desiccation risks might explaining why, although egg survival was highest in the dry area, we found much lower egg batch densities in the dry area compared to the other areas. Our results also suggested that the selection of wetter microhabitats might have enhanced offspring survival by lowering predation from natural enemies. As we found relatively high mortality rates throughout the study area it is possible that there might be other strategies, like using alternative host plants as an enemy free space, through which *G. sagittariae* females could increase offspring survival rates. While the host plant range of *G. sagittariae* includes several plant species [27, 37], we did not observe such behaviour at our field location and it would be of interest to collect long-term data on survival rates among offspring on different host plant across generations to disentangle such a scenario.

## Additional files

**Additional file 1.** Summary of likelihood ratio tests (**Table S1**), and over-view graphs (**Figure S1**) of all variables measured in our study using the area along the soil moisture gradient as explanatory variable.

**Additional file 2.** Summary of the model output for the Random Survival Forest analyses on egg survival and larval survival (**Table S2**) followed by the VIMP ranking (**Figure S2**), the relationship between VIMP and minimal depth ranking (**Figure S3**) and the minimal depth variable interaction plot for the RSF analysis on egg survival (**Figure S4**) and larval survival (**Figure S5**).

**Additional file 3.** Overview table of the natural enemies collected by sweep netting, sticky traps or pitfall traps (**Table S3**) and the parasitism rates of *Asecodes lucens* (**Figure S6**).

## Abbreviations

GLM: generalized linear model
NMDS: non-metric multidimensional scaling
OOB: out-of-bag
RSF: random survival forest
VIMP: variable importance
