# Supplementary material for: A random survival forest illustrates the importance of natural enemies compared to host plant quality on leaf beetle survival rates
Source: BMC Ecol. 2018 Sep 10;18:33. doi: 10.1186/s12898-018-0187-7 (PMC6131828; doi:10.1186/s12898-018-0187-7)
Supplement: Supplementary file 1 — Additional file 1. Summary of likelihood ratio tests (Table S1), and over-view graphs (Figure S1) of all variables measured in our study using the area along the soil moisture gradient as explanatory variable. [file 12898_2018_187_MOESM1_ESM.pdf]

# A random survival forest illustrates the importance of natural enemies compared to host plant quality on leaf beetle survival rates

Additional file 1


**Author affiliation:**
**Thomas A. Verschut*** (http://orcid.org/0000-0003-0130-6485)
Department of Ecology, Environment and Plant Sciences, Stockholm University, 106 91 Stockholm, Sweden.

**Peter A. Hambäck** (http://orcid.org/0000-0001-6362-6199)
Department of Ecology, Environment and Plant Sciences, Stockholm University, 106 91 Stockholm, Sweden.

***Corresponding author:**
**Thomas A. Verschut**
Department of Ecology, Environment and Plant Sciences, Stockholm University, 106 91 Stockholm, Sweden.
Email: thomas.verschut@su.se Phone: +46(0)8 16 38 49

**Table S1.** Summary of likelihood ratio tests ($\chi^2$) for all variables measured in our study. All results listed under the untransformed data column were included in the random survival forest analyses. The variables for which transformations were necessary to meet parametric model requirements are listed under the transformed data column and were used for Figure S1. Vegetation height accounts for the height of non-host plants surrounding *Potentilla palustris* in the plots. The data set column lists which variables were specific for either of the analysis on the eggs or larval data set.

| | | **Untransformed** | | | **Transformed** | | |
|---|---|---|---|---|---|---|---|
| **Variable** | **Data set** | $\chi^2$ | ***df*** | ***p*** | $\chi^2$ | ***df*** | ***p*** |
| Host plant height (cm) | Eggs | 45.49 | 2,299 | **<0.001** | - | - | - |
| Number of eggs | Eggs | 3.75 | 2,299 | 0.15 | - | - | - |
| Vegetation height (cm) | Both | 456.67 | 2,299 | **<0.001** | - | - | - |
| Host plant density (%) | Both | 36.38 | 2,299 | **<0.001** | 42.72 | 2,299 | **<0.001** |
| Leaf moisture content (%) | Both | 9.06 | 2,299 | **0.01** | 9.75 | 2,299 | **0.008** |
| Nitrogen (%) | Both | 38.26 | 2,299 | **<0.001** | 35.56 | 2,299 | **<0.001** |
| Carbon (%) | Both | 151.32 | 2,299 | **<0.001** | 149.49 | 2,299 | **<0.001** |
| Phosphorus (%) | Both | 84.03 | 2,299 | **<0.001** | 83.01 | 2,299 | **<0.001** |
| Host plant height (cm) | Larvae | 16.02 | 2,299 | **<0.001** | - | - | - |
| Number of larvae | Larvae | 4.67 | 2,299 | 0.09 | 1.92 | 2,299 | 0.38 |

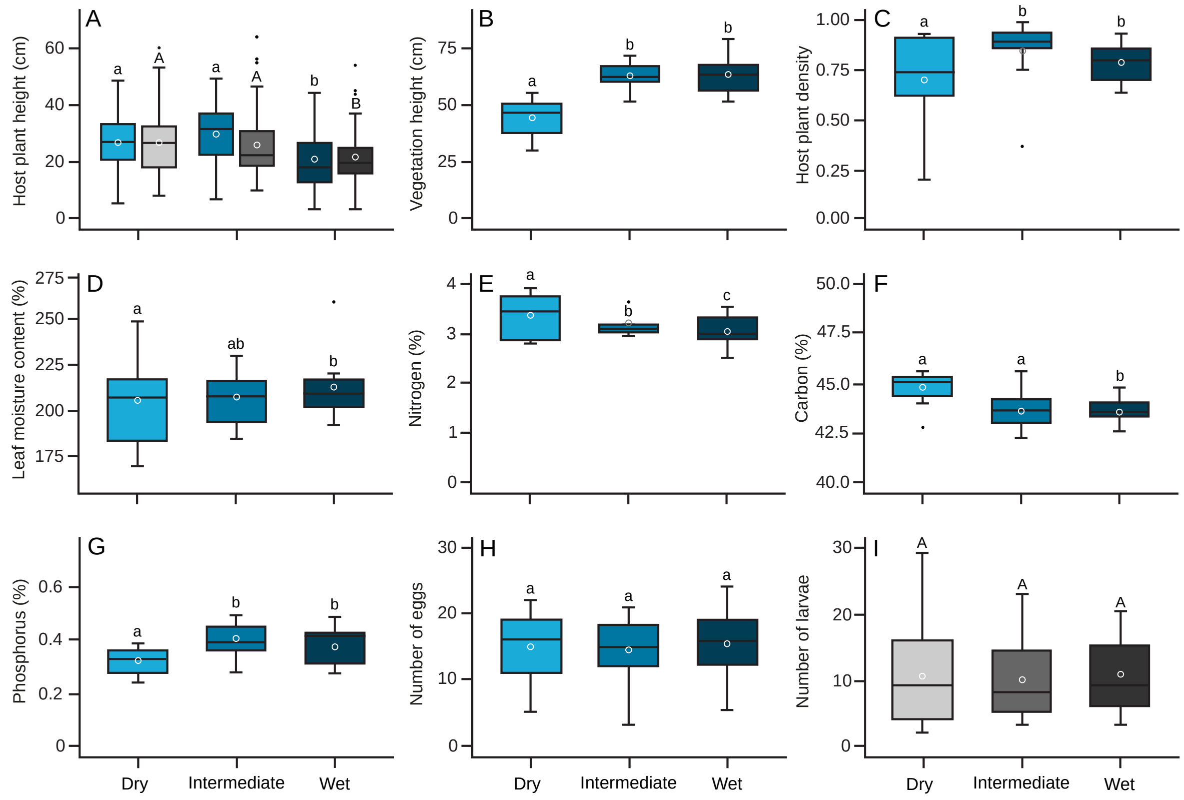


**Figure S1.** Overview of all variables measured in our study using area along the soil moisture gradient (i.e. dry, intermediate and wet) as explanatory variable. The lines within the box plots represent the median and the circle represents the mean. The data specifically used for the random survival forest analysis of the larval data set are given in grey scale. The letters above the error bars indicate statistical differences between the areas and were calculated using Tukey-HSD post hoc analysis with Bonferroni corrections.
