# Supplementary material for: A random survival forest illustrates the importance of natural enemies compared to host plant quality on leaf beetle survival rates
Source: BMC Ecol. 2018 Sep 10;18:33. doi: 10.1186/s12898-018-0187-7 (PMC6131828; doi:10.1186/s12898-018-0187-7)
Supplement: Supplementary file 2 — Additional file 2. Summary of the model output for the Random Survival Forest analyses on egg survival and larval survival (Table S2) followed by the VIMP ranking (Figure S2), the relationship between VIMP and minimal depth ranking (Figure S3) and the minimal depth variable interaction plot for the RSF analysis on egg survival (Figure S4) and larval survival (Figure S5). [file 12898_2018_187_MOESM2_ESM.pdf]

# A random survival forest illustrates the importance of natural enemies compared to host plant quality on leaf beetle survival rates

Additional file 2

**Author affiliation:**
**Thomas A. Verschut*** (http://orcid.org/0000-0003-0130-6485)
Department of Ecology, Environment and Plant Sciences, Stockholm University, 106 91 Stockholm, Sweden.

**Peter A. Hambäck** (http://orcid.org/0000-0001-6362-6199)
Department of Ecology, Environment and Plant Sciences, Stockholm University, 106 91 Stockholm, Sweden.

***Corresponding author:**
**Thomas A. Verschut**
Department of Ecology, Environment and Plant Sciences, Stockholm University, 106 91 Stockholm, Sweden.
Email: thomas.verschut@su.se Phone: +46(0)8 16 38 49

**Table S2.** Summary of the model output for the random survival forest analysis on egg survival and larval survival.

| **Model component** | **Eggs** | **Larvae** |
|---|---|---|
| Sample size | 4504 | 3111 |
| Number of deaths | 1914 | 2877 |
| Number of trees | 1000 | 1000 |
| Minimum terminal node size | 3 | 3 |
| Average no. of terminal nodes | 168.644 | 196.243 |
| No. of variables tried at each split | 3 | 3 |
| Total no. of variables | 9 | 9 |
| Analysis | RSF | RSF |
| Family | Survival | Survival |
| Splitting rule | Random log-rank | Random log-rank |
| Number of random split points | 10 | 10 |
| Out-of-Bag error rate (%) | 12.29 | 42.21 |

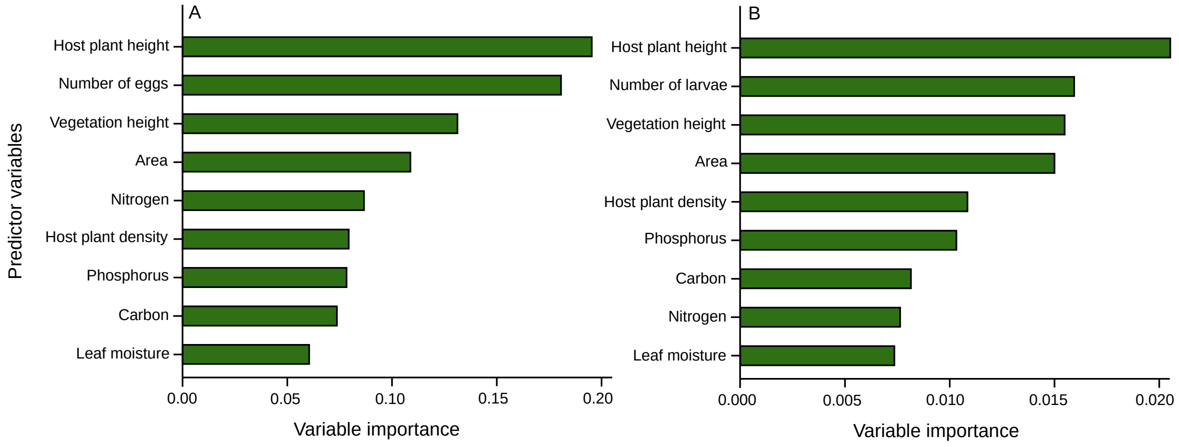


**Figure S2.** Ranking of the predictor variable through variable importance (VIMP) calculated in the random survival forest analysis for (A) egg survival and (B) larval survival. VIMP measures the change in prediction error when the variable of interest would not be available to grow a new forest and the highest ranking variables would lower the predictive strength of the model most when removed.

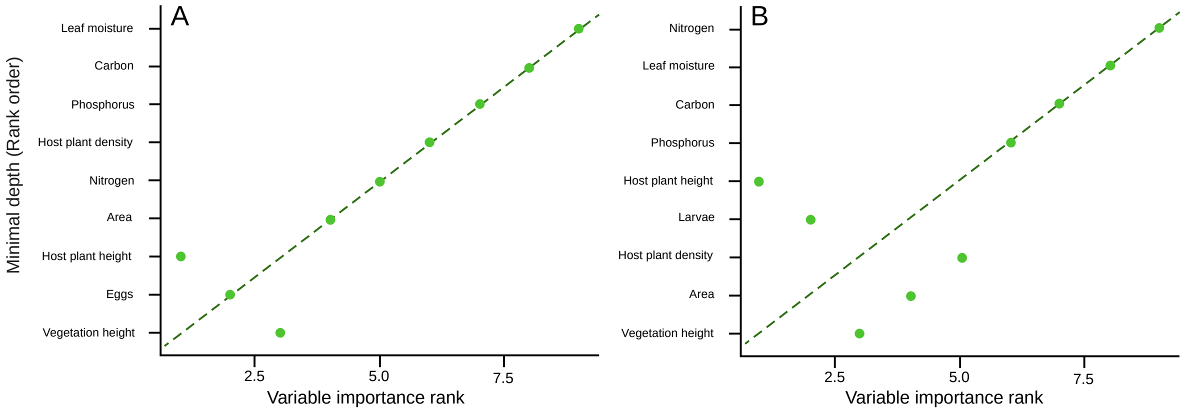


**Figure S3.** Agreement between minimal depth and variable importance rankings for the random survival forest analysis for (A) egg survival and (B) larval survival. Green points on the dark green dashed line indicate variables that are ranked equivalently, points above the dashed line have higher variable importance ranking, while those below the line have higher minimal depth rankings.

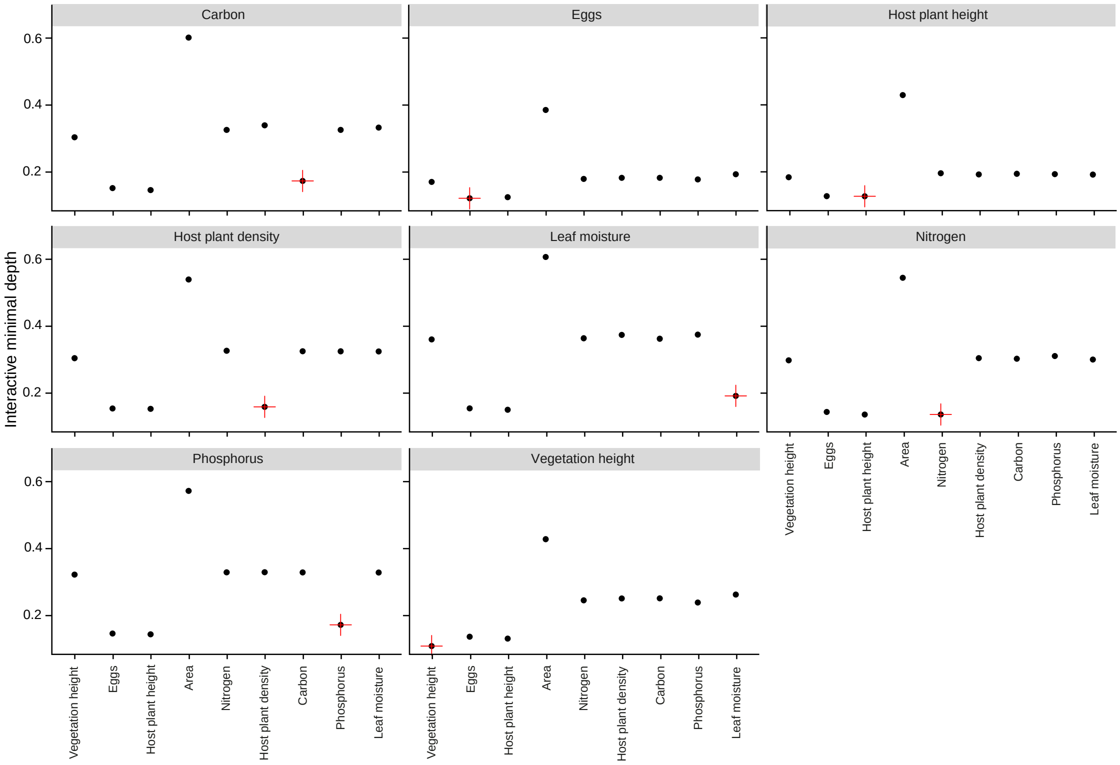


**Figure S4.** Minimal depth variable interaction plot for all variables included in the random survival forest analysis for egg survival. Higher values indicate lower interactivity with the target variable marked by the red cross.

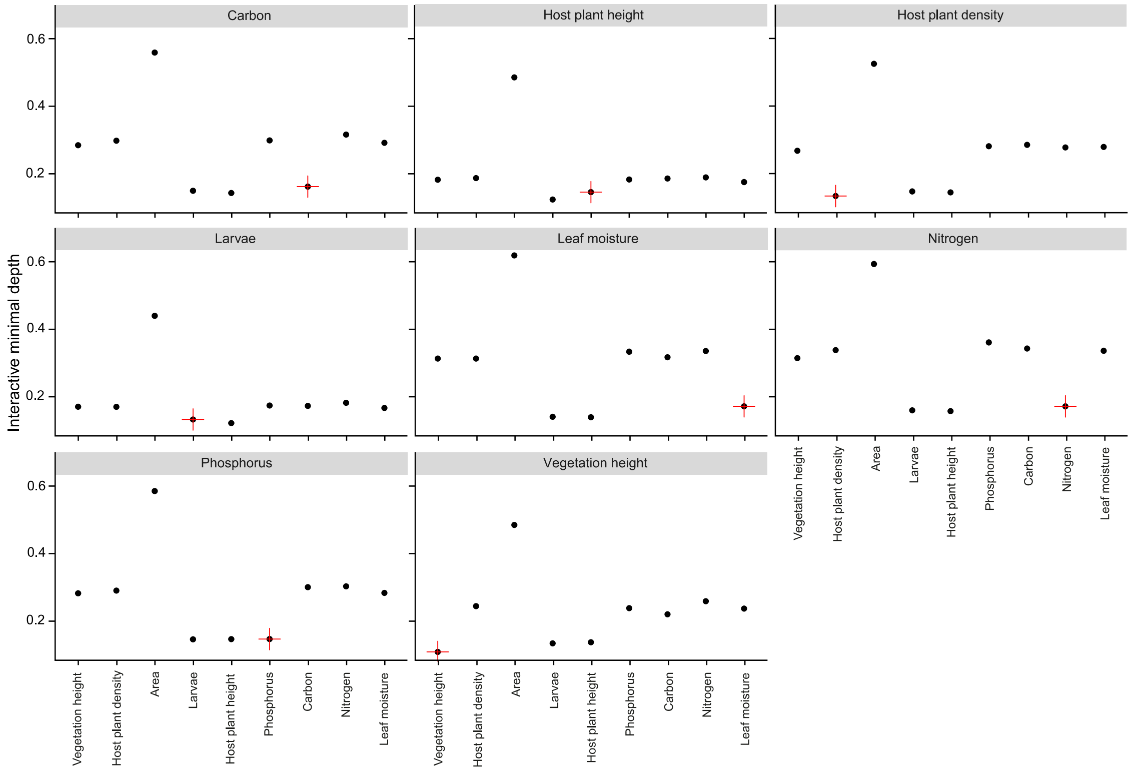


**Figure S5.** Minimal depth variable interaction plot for all variables included in the random survival forest analysis for larval survival. Higher values indicate lower interactivity with the target variable marked by the red cross.
