# Supplementary material for: A random survival forest illustrates the importance of natural enemies compared to host plant quality on leaf beetle survival rates
Source: BMC Ecol. 2018 Sep 10;18:33. doi: 10.1186/s12898-018-0187-7 (PMC6131828; doi:10.1186/s12898-018-0187-7)
Supplement: Supplementary file 3 — Additional file 3. Overview table of the natural enemies collected by sweep netting, sticky traps or pitfall traps (Table S3) and the parasitism rates of Asecodes lucens (Figure S6). [file 12898_2018_187_MOESM3_ESM.pdf]

A random survival forest illustrates the importance of natural enemies compared to host plant quality on leaf beetle survival rates

Additional file 3

Author affiliation:
Thomas A. Verschut* (http://orcid.org/0000-0003-0130-6485)
Department of Ecology, Environment and Plant Sciences, Stockholm University, 106 91 Stockholm, Sweden.

Peter A. Hambäck (http://orcid.org/0000-0001-6362-6199)
Department of Ecology, Environment and Plant Sciences, Stockholm University, 106 91 Stockholm, Sweden.

***Corresponding author:**
Thomas A. Verschut
Department of Ecology, Environment and Plant Sciences, Stockholm University, 106 91 Stockholm, Sweden.
Email: thomas.verschut@su.se Phone: +46(0)8 16 38 49

Table S3. Overview table of all potential natural enemies collected by sweep netting, sticky traps or pitfall traps. All species of which we only collected five or less individuals were not included in the statistical analysis and are indicated with an asterisk (*). For *Asecodes lucens* we give the average parasitism rate in five plots per area rather than caught individuals. Additional data on the parasitism rates can be found in figure S6.

		Dry			Intermediate			Wet		
	**Species**	**Sweep**	**Sticky**	**Pitfall**	**Sweep**	**Sticky**	**Pitfall**	**Sweep**	**Sticky**	**Pitfall**
**Araneae**	**Lycosidae**									
	*Acantholycosa lignaria**	-	-	1	-	-	-	-	-	-
	Pardosa fulvipes	-	-	17	-	-	1	-	-	-
	Pardosa lugubris	-	-	5	-	-	-	-	-	-
	Pardosa prativaga	-	-	40	-	-	34	1	-	36
	*Pardosa pullata**	-	-	3	-	-	-	-	-	-
	Pirata piraticus	-	-	51	-	-	28	1	-	24
	Pirata piscatorius	-	-	-	-	-	10	-	-	11
	Piratula uliginosa	-	-	7	-	-	1	-	-	-
	Trochosa ruricola	-	-	1	-	-	2	-	-	3
	**Pisauridae**									
	Dolomedes fimbriatus	14	-	-	3	-	1	1	-	-
	**Salticidae**									
	*Marpissa radiate**	1	-	-	1	-	-	-	-	-
	**Theridiidae**									
	*Neottiura bimaculata**	2	-	-	-	-	-	-	-	-
	Phylloneta impressa	-	-	-	5	-	-	1	-	-
	Theridion varians	2	-	-	3	-	-	-	-	1
	*Theridiosoma gemmosum**	-	-	-	-	-	-	-	-	1
**Coleoptera**	**Cantharidae**									
	Crudosilis ruficollis	-	-	-	-	3	-	2	5	-
	Podistra rufotestacea	8	5	-	4	1	-	6	2	-
	**Carabidae**									
	Agonum fuliginosum	-	-	5	-	-	3	-	-	-
	*Agonum micans**	-	-	1	-	-	-	-	-	-
	Agonum versutum	-	-	-	-	-	11	-	-	10
	Elaphrus cupreus	-	-	-	-	-	-	-	-	8
	Loricera pilicornis	-	-	4	-	-	9	-	-	6
	Oodes helopioides	-	-	2	-	-	19	-	-	17
	Pterostichus anthracinus	-	-	-	-	-	12	-	-	14
	*Pterostichus melanarius**	-	-	2	-	-	1	-	-	-
	Pterostichus minor	-	-	14	-	-	6	-	-	28
	Pterostichus nigrita	-	-	11	-	-	17	-	-	29
	Pterostichus oblongopunctatus	-	-	-	-	-	2	-	-	6
	**Carabus**									
	Carabus granulatus	-	-	8	-	-	5	-	-	12
	**Coccinellidae**									
	*Propylea quatuordecimpunctata**	-	1	-	1	-	-	1	-	-
	Anisosticta novemdecimpunctata	-	-	-	8	1	-	28	8	-
	Adalia bipunctata	-	-	-	3	-	-	11	-	-
	Coccinella hieroglyphica	-	-	-	1	-	-	9	3	-
	*Coccinella septempunctata**	-	-	-	-	-	-	1	-	-
	**Hydrophilidae**									
	*Hydrochara caraboides**	-	-	1	-	-	-	-	-	-
	**Staphylinidae**									
	Aleochara brevipennis	-	2	12	-	-	3	-	2	14
	*Lathrobium elongatum**	-	-	-	-	-	1	-	-	1
	Paederus riparius	-	-	-	3	5	17	2	1	3
	Philonthus addendus	-	-	-	-	-	-	-	-	7
	*Philonthus atratus**	-	-	-	-	-	-	-	-	3
	*Philonthus corvinus**	-	-	-	-	-	2	-	-	-
	Philonthus fumarius	-	-	4	-	-	4	-	2	11
	*Philonthus marginatus**	-	-	2	-	-	-	-	-	-
	*Philonthus micans**	-	-	1	-	-	2	-	-	-
	*Philonthus succicola**	-	-	1	-	-	-	-	-	-
	Quedius fuliginosus	-	-	11	-	2	19	-	-	21

	Tachinus rufipes	-	-	5	-	-	-	-	-	-
	*Zyras collaris**	-	-	3	-	-	-	-	-	-
**Hymenoptera**	**Formicidae**									
	Formica polyctena	-	-	648	-	-	14	-	-	-
	Myrmica ruginodis	-	-	42	-	-	-	-	-	-
	*Camponotus ligniperda**	-	-	-	-	-	2	-	-	1
	**Eulophidae**									
	*Asecodes lucens**	9.17 ± 4.97%			15.56 ± 8.64%			19.49 + 4.23%		

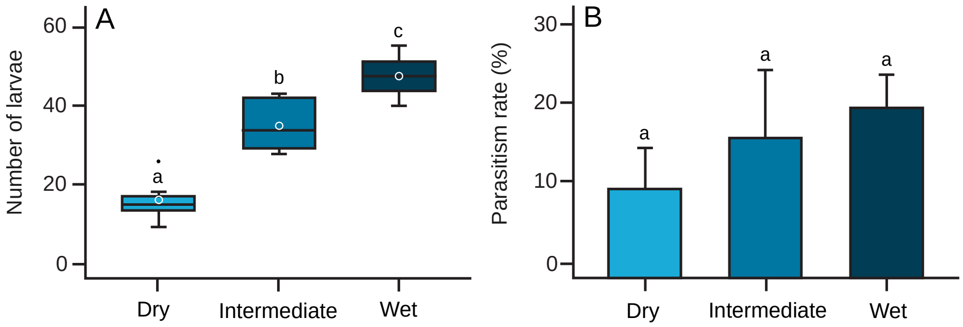

Figure S6. (A) The average number of surviving larvae in five plots in the different areas along the soil moisture gradient, and (B) the corresponding parasitism rate by *Asecodes lucens*. The lines within the box plots represents the median and the circle represents the mean. The letters above the error bars indicate statistical differences between the areas and were calculated using Tukey-HSD post hoc analysis with Bonferroni corrections.
